# Massively Parallel Coincidence Counting of High-Dimensional Entangled States

**DOI:** 10.1038/s41598-018-26144-7

**Published:** 2018-05-21

**Authors:** Matthew Reichert, Hugo Defienne, Jason W. Fleischer

**Affiliations:** Department of Electrical Engineering, Princeton University, Princeton, NJ 08544 USA

## Abstract

Entangled states of light are essential for quantum technologies and fundamental tests of physics. Current systems rely on entanglement in 2D degrees of freedom, e.g., polarization states. Increasing the dimensionality provides exponential speed-up of quantum computation, enhances the channel capacity and security of quantum communication protocols, and enables quantum imaging; unfortunately, characterizing high-dimensional entanglement of even bipartite quantum states remains prohibitively time-consuming. Here, we develop and experimentally demonstrate a new theory of camera detection that leverages the massive parallelization inherent in an array of pixels. We show that a megapixel array, for example, can measure a joint Hilbert space of 10^12^ dimensions, with a speed-up of nearly four orders-of-magnitude over traditional methods. The technique uses standard geometry with existing technology, thus removing barriers of entry to quantum imaging experiments, generalizes readily to arbitrary numbers of entangled photons, and opens previously inaccessible regimes of high-dimensional quantum optics.

## Introduction

Broad beams of quantum light are a natural pathway to large Hilbert spaces^1–11^, as they have high-dimensional entanglement in transverse spatial modes^12^. Spatial correlation of biphotons has led to sub-shot-noise quantum imaging^13,14^, enhanced resolution^15^, quantum ghost imaging^16^, and proposals for quantum lithography^17^. Despite this work, high-dimensional quantum optics remains underdeveloped, largely due to difficulty in measuring the full joint probability distribution. Traditionally, experiments measure coincidences between two single-photon counting modules (SPCMs) that are each scanned over their own subspace to build up a measurement point-by-point. Such a procedure is photon-inefficient, making high-dimensional measurements tedious and prohibitively time consuming. Full quantum-state measurements are impractical even for a relatively small number of dimensions^18,19^.

In this work, we present a rapid and efficient method of measuring a high-dimensional biphoton joint probability distribution via massively parallel coincidence counting. We use a single-photon-sensitive electron-multiplying (EM) CCD camera as a dense array of photon detectors to measure all dimensions of the joint Hilbert space simultaneously. For example, a typical megapixel camera can record a one trillion-dimensional joint Hilbert space nearly 10,000× faster than traditional raster-scanning methods. This speed-up enables observation of high-dimensional features that cannot be seen when only low-dimensional measurements (projections) are made.

Recent efforts with single-photon-sensitive cameras have characterized spatial entanglement^20–25^, but results relied on projection onto only two dimensions and considered only homogeneous distributions, limiting measurements to EPR-type entanglement. In these works, measurements were spatially averaged over the entire plane (losing local detail). Further, to mitigate complications of accidental counts, coincidence measurements were performed in the low-count-rate regime. Here, we show that this assumption is unnecessary and give a general expression for the biphoton joint probability distribution. The exact expression follows from measurements of single- and coincidence-count probabilities observed between every pair of pixels over the entire frame simultaneously. The resulting distribution is valid for arbitrary count rates up to detector saturation, enabling more accurate measurements, faster acquisition speeds, and optimization of the signal-to-noise ratio.

To demonstrate our method, we characterize the properties of photon pairs entangled in transverse spatial degrees of freedom. A pure entangled photon state is described by the biphoton wave function $\psi ({{\boldsymbol{\rho }}}_{i},{{\boldsymbol{\rho }}}_{j})$, where ${{\boldsymbol{\rho }}}_{i}={x}_{i}{\hat{{\bf{x}}}}_{i}+{y}_{i}{\hat{{\bf{y}}}}_{i}$, and likewise for ${{\boldsymbol{\rho }}}_{j}$. The joint probability of observing one photon at ${{\boldsymbol{\rho }}}_{i}$ and its partner at ${{\boldsymbol{\rho }}}_{j}$ is ${\rm{\Gamma }}({{\boldsymbol{\rho }}}_{i},{{\boldsymbol{\rho }}}_{j})={|\psi ({{\boldsymbol{\rho }}}_{i},{{\boldsymbol{\rho }}}_{j})|}^{2}$, which in a discretized basis is ${{\rm{\Gamma }}}_{ij}$. Since each photon may be found in a 2D space (*x*_*i*_, *y*_*i*_), the joint probability distribution is a 4D distribution. Like classical light-field methods^26^, observation of the full 4D distribution shows details and features that would be lost with conventional projection methods. While we focus on spatial components, we emphasize that our technique may be readily extended to other degrees of freedom, such as spectral modes or orbital angular momentum, by suitable mapping onto the pixels of the camera.

**Figure 1:**
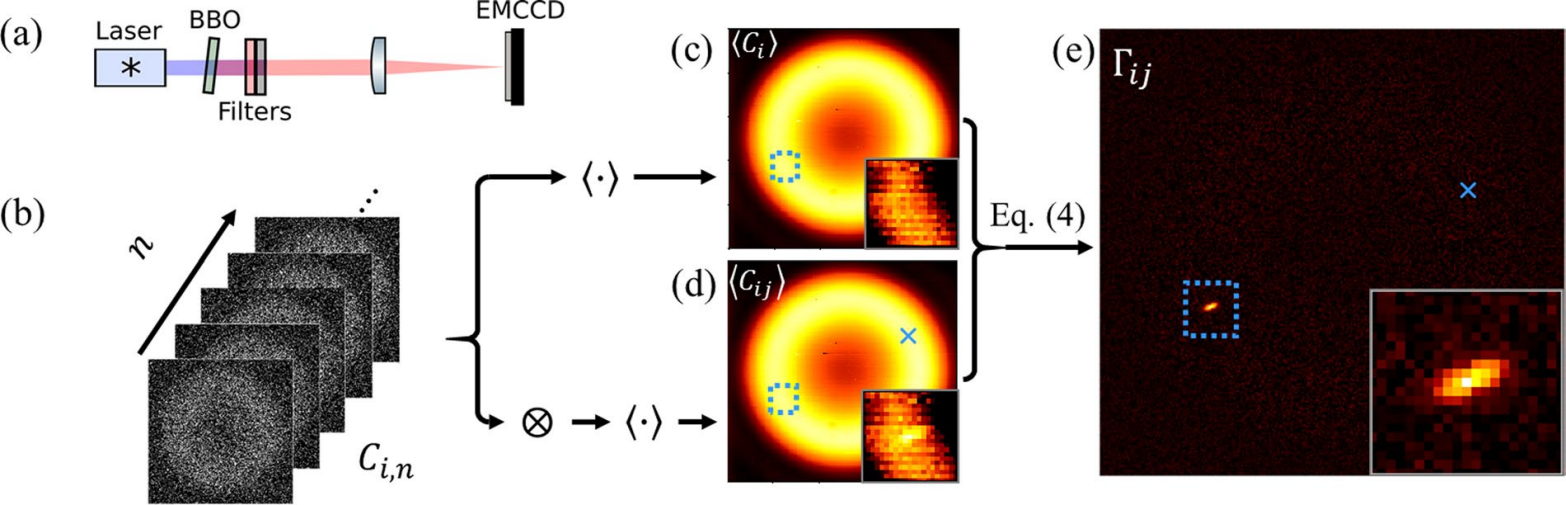
Measuring the biphoton joint probability distribution with an EMCCD camera. (**a**) Experimental setup for measuring far-field type-I SPDC. (**b**–**e**) Flow chart of data processing. (**b**) The camera acquires many thresholded frames from which we calculate both (**c**) the average of all frames 〈${C}_{i}$〉 (indicated by 〈·〉) and (**d**) the average of the tensor product of each frame with itself 〈${C}_{ij}$〉 (⊗, Eq. (2)) (shown here for $j=[{x}_{j}=70,\,{y}_{j}=33]$, indicated by the blue ×). Most coincidences are accidentals between photons from different pairs, yielding the apparent similarity between (**c**) and (**d**). Genuine coincidences from anticorrelated entangled photons appearing within the boxed region give a difference between the two (see insets). (**e**) The conditional probability distribution, via Eq. (4), shows anti-correlation of paired photons localized about $i$ = [−70, −32].

A schematic of the measurement and processing procedure is shown in Fig. 1. Spatially entangled photon pairs are generated via spontaneous parametric down-conversion (SPDC) in a β-barium borate (BBO) crystal, cut for type-I phase matching. The spatial entanglement structure has been extensively studied^12,13,15,21,24,25,27–33^, and we use it here for a clear experimental demonstration of high-dimensional characteristics of entangled photons. The crystal is pumped by a 120 mW, 400 nm cw laser diode that is spatially filtered and collimated (not shown). Spectral filters block the pump beam and select near-degenerate photon pairs at 800 nm (a large bandwidth of 40 nm (FWHM), gives rise to the relatively thick rings in the far field^32,33^). These are placed immediately after the BBO crystal to prevent induced fluorescence in the subsequent optics. A lens images the far field of the crystal onto an EMCCD camera (Andor iXon Ultra).

Measurement of the biphoton joint probability distribution ${{\rm{\Gamma }}}_{ij}$ is possible with an EMCCD camera due to its high quantum efficiency and low noise floor. The camera is operated in the photon-counting regime, where each pixel is set to one if its gray-level output is above a threshold and zero otherwise^34^ (see Methods). The data consist of a set of $N$ frames ${C}_{i,n}$ = {0, 1}, where subscript $i$ is the pixel index (spatial mode) and $n$ is the frame number. Each frame consists of many counts from both photon events and electronic noise (mainly due to clock-induced charge^34^). The singles-count probability is

1 $$\langle {C}_{i}\rangle =\sum _{m}{P}_{m}({\mu }_{i|m}+{p}_{el}{\mu }_{\bar{i}|m}),$$

where ${P}_{m}$ is the distribution of the number $m$ of photon pairs and ${p}_{el}$ is the electronic count probability (e.g., dark counts). The factors ${\mu }_{i|m}$ and ${\mu }_{\bar{i}|m}$ represent the conditional probabilities of detecting *at least* one photon and zero photons, respectively, given $m$ pairs arriving within the detector time window (see Table 1)^35^.

**Table 1:** Probabilities of single detection ${\mu }_{p|m}$ and coincidence ${\mu }_{pq|m}$ conditioned on the number of photon pairs *m*.

Term	Expression
${\mu }_{i|m}$	$1-{\mu }_{\bar{i}|m}$
${\mu }_{\bar{i}|m}$	${(1-2\eta {{\rm{\Gamma }}}_{i}+{\eta }^{2}{{\rm{\Gamma }}}_{ii})}^{m}$
${\mu }_{ij|m}$	$1-{\mu }_{\bar{i}|m}-{\mu }_{\bar{j}|m}+{\mu }_{\bar{i}\bar{j}|m}$
${\mu }_{i\bar{j}|m}$	${\mu }_{\bar{j}|m}-{\mu }_{\bar{i}\bar{j}|m}$
${\mu }_{\bar{i}\bar{j}|m}$	${(1-2\eta ({{\rm{\Gamma }}}_{i}+{{\rm{\Gamma }}}_{j})+{\eta }^{2}({{\rm{\Gamma }}}_{ii}+{{\rm{\Gamma }}}_{jj}+2{{\rm{\Gamma }}}_{ij}))}^{m}$

Γ_*pq*_ is the joint probability distribution, Γ_*p*_ is the marginal, and *η* is the detection quantum efficiency. Barred subscript indicates no detection.

Since the duration of both the exposure and read-out of each frame of the EMCCD is much longer than the biphoton correlation time, photons from each pair arrive at the camera within a single frame. The coincidence count probability between all pixels *i* and *j* is measured by the average of the tensor product of each frame with itself:

2 $$\langle {C}_{ij}\rangle =\frac{1}{N}\sum _{n=1}^{N}{C}_{i,n}{C}_{j,n}.$$

In addition to genuine coincidence counts from entangled photon pairs, there are also accidental counts from uncorrelated photons and noise. These can be accounted for in general by the expression

3 $$\langle {C}_{ij}\rangle =\sum _{m}{P}_{m}({\mu }_{ij|m}+{p}_{el}({\mu }_{i\bar{j}|m}+{\mu }_{\bar{i}j|m})+{p}_{el}^{2}{\mu }_{\bar{i}\bar{j}|m}),$$

Where each of the terms ${\mu }_{pq|m}$ are related to ${{\rm{\Gamma }}}_{pq}$ and its marginal (see Table 1). The terms in Eq. (3) are coincidences between (1) *at least* two photons, (2) *at least* one photon and one electronic noise event, and (3) two noise events. For a Poissonian distribution of pairs, Eq. (3) simplifies, giving an analytic expression for $\langle {C}_{ij}\rangle$ in terms of $\langle {C}_{i}\rangle$, $\langle {C}_{j}\rangle$, and ${{\rm{\Gamma }}}_{ij}$. With Eq. (1) this yields

4 $${{\rm{\Gamma }}}_{ij}=\alpha \,{\rm{l}}{\rm{n}}(1+\frac{\langle {C}_{ij}\rangle -\langle {C}_{i}\rangle \langle {C}_{j}\rangle }{(1-\langle {C}_{i}\rangle )(1-\langle {C}_{j}\rangle )})$$

where *α* is a constant that depends on the quantum efficiency of the system (see Supplementary Information).

Equation (4) includes the case when several photons arrive at the same pixel. This case has been excluded explicitly by other treatments^21,22,35^, even though collinear geometry and high spatial entanglement make this case the most likely one. The paradox is often circumvented by considering the low-photon-count limit, in which the joint probability distribution ${{\rm{\Gamma }}}_{ij}$ becomes proportional to the measured coincidence count rate $\langle {C}_{ij}\rangle$. However, this assumption is not necessary here; indeed, Eq. (4) remains valid up to detector saturation. The formalism thus covers the entire range of photon intensities and types of detection events, and generalizes straightforwardly to joint distributions of higher numbers of entangled photons.

Figure 1d shows the coincidence count distribution for a particular pixel $j$ = [${x}_{j}$ = 70, ${y}_{j}$ = 33], i.e., a 2D slice for all $i$ = {${x}_{i}$, ${y}_{i}$} through the 4D joint distribution $\langle {C}_{ij}\rangle$. It includes genuine coincidences as well as a large background from accidental counts. Due to the large number of pairs in each frame (~10^4^), most coincidences are accidentals between photons from different pairs; indeed, Fig. 1d appears very similar to the singles count distribution $\langle {C}_{i}\rangle$ in Fig. 1c. Genuine coincidences between photons from the same pair, shown in the inset, rise above the background from accidentals. The corresponding 2D slice through the 4D ${{\rm{\Gamma }}}_{ij}$, calculated via Eq. (4), is displayed in Fig. 1e. When one photon is found at $j$ = [70, 33], its entangled partner is localized near $i$ = [−70, −32], indicating a high degree of anti-correlation. Such conditional distributions ${{\rm{\Gamma }}}_{i|j}$ are measured simultaneously for all $j$, thus constituting a full measurement of the 4D biphoton joint probability distribution.

**Figure 2:**
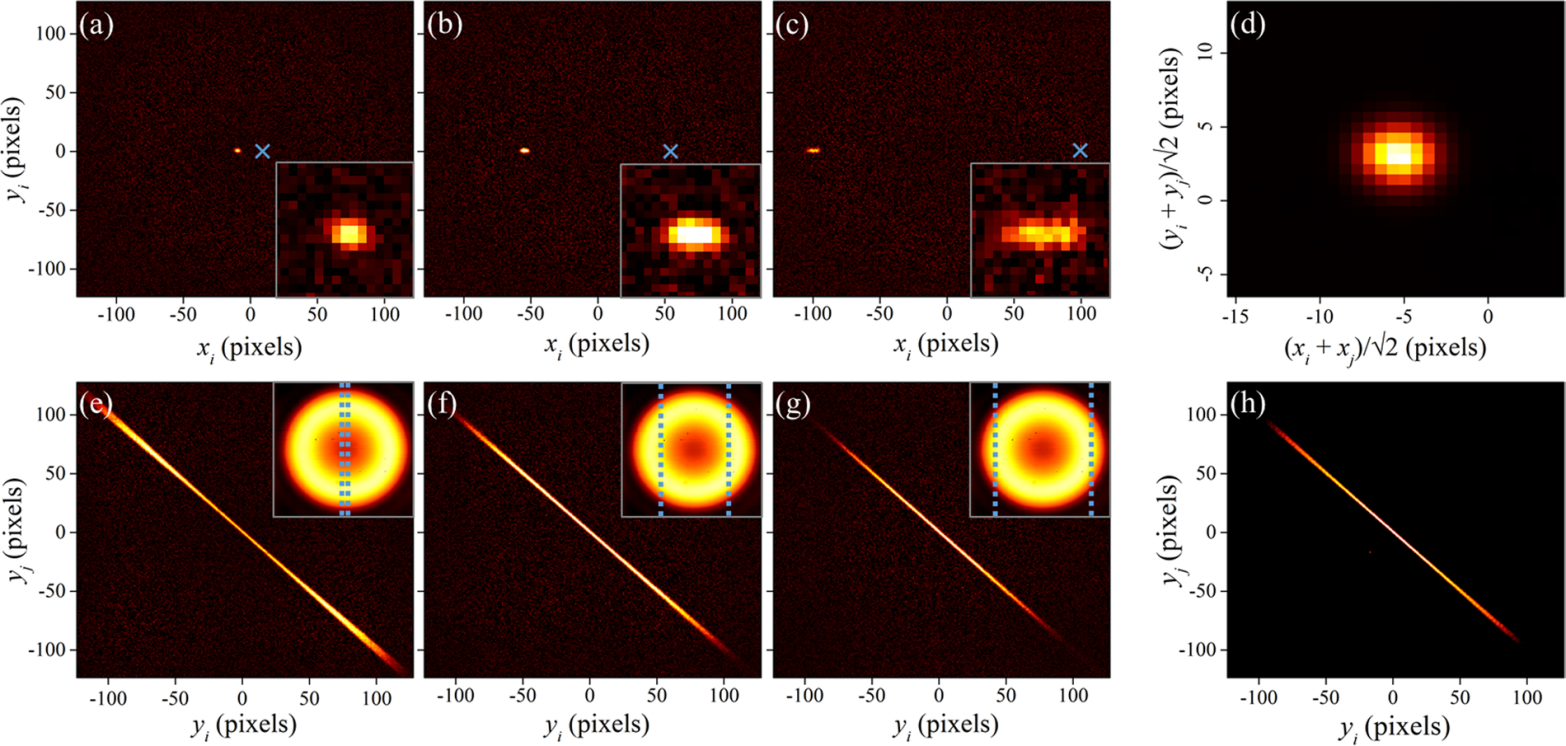
Information contained in the full 4D measurement of biphoton joint probability distribution. (**a**–**c**) Variation of ${{\rm{\Gamma }}}_{i|j}$ at different distances from the center, indicated by blue ×, showing anti-correlation of width that increases with |*x*| (see insets). (**d**) Projection of ${{\rm{\Gamma }}}_{ij}$ onto sum coordinates averages the variations in (**a**–**c**). (**e**–**g**) 2D slices of ${{\rm{\Gamma }}}_{ij}$ for fixed $[{x}_{i},\,{x}_{j}]$ (indicated by blue dashed lines in inset of 〈${C}_{i}$〉) showing variation in anti-correlation with horizontal separation. (**h**) Projection of ${{\rm{\Gamma }}}_{ij}$ onto $[{y}_{i},\,{y}_{j}]$ (integration over ${x}_{i}$ and ${x}_{j}$) averages the structures in (**e**–**g**), giving only a mean profile.

Complete measurements of high-dimensional joint Hilbert spaces contain detailed, localized information not available in lower-dimensional projections. To demonstrate this, we show ${{\rm{\Gamma }}}_{i|j}$ for entangled photons detected at different radial distances $j$ = [*x*_*j*_, *y*_*j*_] from the center of the beam (Fig. 2a–c). There are two main observations: 1) as *x*_*j*_ increases, *x*_*i*_ decreases, and 2) the width along the radial directions increases. The former is necessary to maintain a fixed sum, i.e., ${x}_{i}$ + ${x}_{j}$ ≈ 0, while the latter arises from the radial dependence of the uncertainty in the wave vector **k**, ${\rm{\Delta }}{k}_{\rho }\approx {k}_{\rho }|{\rm{\Delta }}{\bf{k}}|/|{\bf{k}}|$. This effect comes from the rather large spectral bandwidth of the filter (40 nm), as different frequencies are phase-matched at different radial momenta ${k}_{\rho }$^27,33^. Observation of such features with traditional raster-scanning techniques requires multiple separate measurements. With an EMCCD camera, they are all captured simultaneously in a single image.

In previous studies, the intercorrelation function was measured via image correlation techniques^21,22^, without measuring the full 4D ${{\rm{\Gamma }}}_{ij}$. However, such measurements provide only the globally averaged correlation and thus neglect any potential internal variation in the joint probability distribution. Here, we calculate the intercorrelation function by projecting ${{\rm{\Gamma }}}_{ij}$ onto the sum coordinate $[({x}_{i}+{x}_{j})/\sqrt{2},({y}_{i}+{y}_{j})/\sqrt{2}]$ (Fig. 2d). The peak near the center indicates that entangled photon pairs are always found near equal and opposite sides of the center, within anti-correlation widths ${\sigma }_{x,+}$ = 20.9 ± 0.3 μm and ${\sigma }_{y,+}$ = 18.6 ± 0.3 μm. Our more-resolved methods show that, even in this simple case, the corresponding widths of the ${{\rm{\Gamma }}}_{i|j}$ in Fig. 2a–c vary significantly, with ${\sigma }_{x}$ = 16.1 ± 1.4 μm, 23.0 ± 1.5 μm, and 34.9 ± 2.5 μm, respectively.

Other slices of ${{\rm{\Gamma }}}_{ij}$, along different coordinates, contain different information about the entangled photon pairs. For example, we examine correlations in vertical position within specific columns of the image by fixing [*x*_*i*_, *x*_j_]. While some variation can survive averaging by projection onto 2D planes, such as phase-matching and spatial walk-off effects (as observed in type II SPDC^28^), in contrast our method is capable of measuring arbitrary 4D joint probability distributions. Examples in Fig. 2e–g show strong vertical anti-correlation that changes depending on the horizontal separation of the selected columns (indicated in the insets), with radial variation that diminishes for larger $|x|$. As before, projecting ${{\rm{\Gamma }}}_{ij}$ averages this variation (Fig. 2h), resulting in lost information^22,23,28^.

The massively parallel capability of EMCCD cameras allows for much faster measurement of ${{\rm{\Gamma }}}_{ij}$ than traditional scanning techniques. Raster-scanning pairs of SPCMs, each in a $d$-dimensional plane, requires ${d}^{2}$ measurements to build a complete measurement. In contrast, an EMCCD measures the entire plane at once, with pixels at each point in the array. While SPCMs have a high effective frame rate (10s of MHz), the frame rate of an EMCCD camera is limited by the readout process (which scales as $\sqrt{d}\,$^36^). Data shown in Figs. 1 and 2 were taken from a subset of 251 × 251 pixels, corresponding to a four-billion-dimensional joint Hilbert space, and were acquired in a matter of hours. A megapixel EMCCD can record a (1024 × 1024)^2^ ≈ one trillion dimensional joint Hilbert space with a signal-to-noise ratio of 10 in approximately 11 hours. The same measurement performed with raster-scanning SPCMs is estimated to take 9 years, giving a camera improvement of ~7000×. The EMCCD camera also outperforms compressive sensing methods^29^ for large joint Hilbert spaces and does not require sparsity or numerical retrieval.

**Figure 3:**
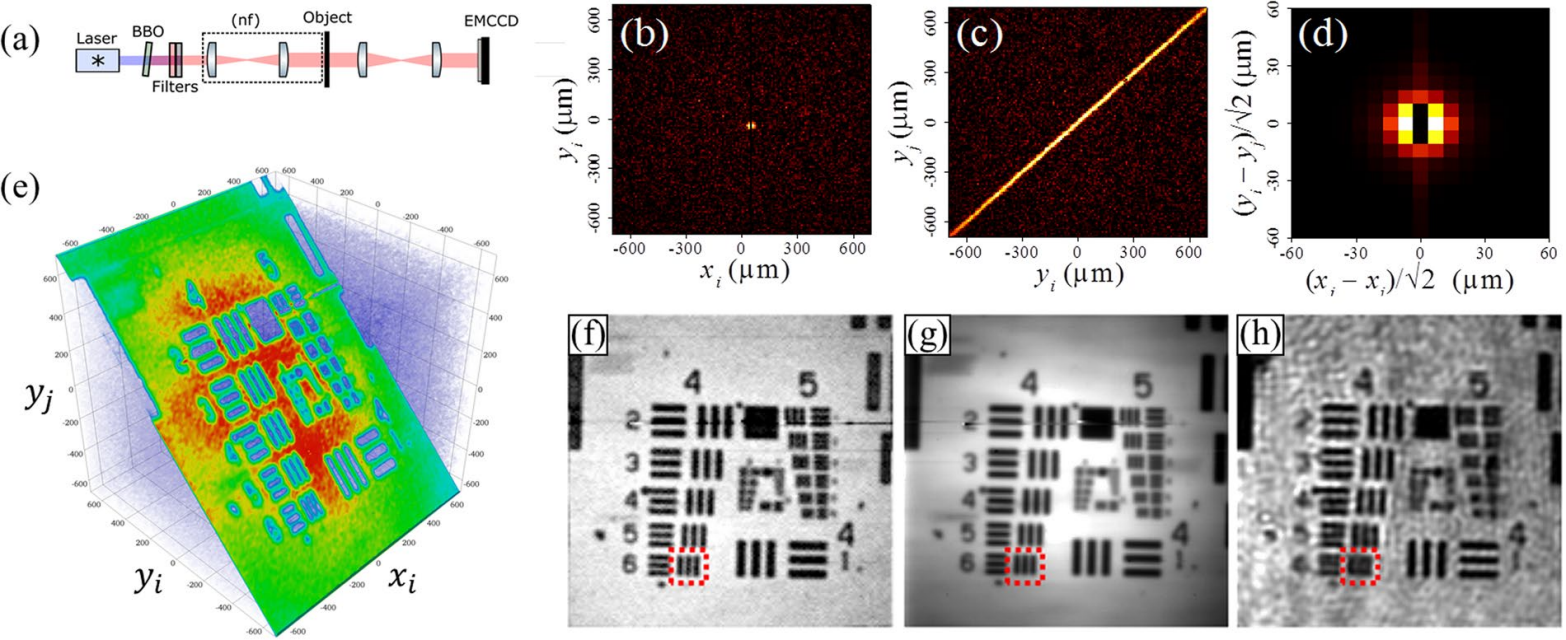
Biphoton imaging of a USAF resolution chart with an EMCCD camera (a) Experimental setup for imaging with the near-field of the biphoton distribution. (**b**–**d**) Measurements of incident ${{\rm{\Gamma }}}_{ij}$ (without the object), showing (**b**) ${{\rm{\Gamma }}}_{i|j}$ for $j=[{x}_{i}=50\,\mathrm{\mu m},{y}_{i}=-40\,\mathrm{\mu m}]$, (**c**) 2D slice of ${{\rm{\Gamma }}}_{ij}$ for fixed $[{x}_{i},\,{x}_{j}]$, and (**d**) projection onto the difference coordinates. Each shows a high degree of spatial correlation. Black region ${x}_{j}={x}_{i}$ in (**b**,**d**) results from zeroing to eliminate the artifact from charge transfer inefficiency (see Methods and Supplementary Information). (**e**) 3D projection of ${{\rm{\Gamma }}}_{ij}$ onto $({x}_{i},\,{y}_{i},\,{y}_{j})$, shows both the image of the resolution chart and spatial correlation of the entangled photons. (**f**–**h**) Comparison of imaging (**f**) Γ_*ij*_ and (**g**) Γ_*i*_ (via singles counts) of entangled photon pairs at 800 nm and (**h**) classical coherent light at 808 nm. Red boxed highlights enhanced in visibility of group 4, element 6.

Camera-based methods hold clear advantages for quantum imaging applications. Imaging with perfectly correlated photon pairs—with biphoton wave function $\psi ({{\boldsymbol{\rho }}}_{i},{{\boldsymbol{\rho }}}_{j})=\delta ({{\boldsymbol{\rho }}}_{i}-{{\boldsymbol{\rho }}}_{j})$—gives a probability distribution of both photons at the same position in the image plane

5 $${\rm{\Gamma }}({\boldsymbol{\rho }},{\boldsymbol{\rho }})\propto {|{\int }^{}{t}^{2}({\boldsymbol{\rho }}{\boldsymbol{^{\prime} }}){h}^{2}({\boldsymbol{\rho }}-{\boldsymbol{\rho }}{\boldsymbol{^{\prime} }}){\rm{d}}{\boldsymbol{\rho }}{\boldsymbol{^{\prime} }}|}^{2}$$

where $t({\boldsymbol{\rho }})$ is the object transmittance and $h({\boldsymbol{\rho }})$ is the point spread function. The fact that the square of $h({\boldsymbol{\rho }})$ appears in Eq. (5) means that biphoton imaging has higher resolution than classical coherent imaging [though it has the same resolution as classical incoherent light (of the same coherence area)^17,30,31,37,38^]. To demonstrate this, we image a resolution chart using spatially entangled biphoton illumination—where one photon is localized near its partner ($i$ ≈ $j$)—by projecting the output facet of the nonlinear crystal onto the object, which is then imaged onto the camera (see Fig. 3a, Methods). To ensure the validity of Eq. (5), we measure the incident ${{\rm{\Gamma }}}_{ij}$ without the object; the results confirm strong spatial correlation, visible in both the conditional distributions (Fig. 3b,c) and the projection onto the difference coordinates (Fig. 3d). By fitting to a Gaussian distribution, we find the correlation width ${\sigma }_{-}$ = 8.5 ± 0.5 μm. Measurements are then repeated with the object; a 3D projection of ${{\rm{\Gamma }}}_{ij}$, shown in Fig. 3e, displays the image of the resolution chart, its appropriate basis (diagonal plane), and the final spatial correlation distribution of the biphotons (thickness of the diagonal plane). Furthermore, coincidence images taken with entangled photon pairs (Fig. 3f) show nearly identical resolution as incoherent light^17,30,31^—as measured by direct imaging (singles counts) of photon pair illumination—(Fig. 3g), and clear improvement in resolution over those with an 808 nm laser diode (Fig. 3h), with less noise and higher visibility. For example, the bars within the red boxed region (group 4, element 6) are clearly resolved with entangled photon pairs (${{\rm{\Gamma }}}_{ii}$, visibility of 0.33 ± 0.03) and incoherent light (${{\rm{\Gamma }}}_{i}$, visibility of 0.37 ± 0.03), but not with classical coherent light (visibility < 0.04). Ideally, the visibility for entangled photon pairs and incoherent light should be the same; the discrepancy here may be due to the way we approximate ${{\rm{\Gamma }}}_{ii}$ with ${{\rm{\Gamma }}}_{i,i+1}\,$using adjacent pixels (see Methods).

By using readily available technology and standard imaging geometries, our method removes barriers of entry to experiments in quantum optics. Time-resolved measurements of coincidence counts are replaced by time-averaged camera measurements of photon correlations, while lower-order counts and conditional probabilities are bootstrapped to provide complete characterization of joint distribution functions. Further, the massive parallelization inherent in megapixel cameras enables measurement of states with orders-of-magnitude greater dimensionality than previously possible, with similar increases in acquisition speed. With suitable mapping for other degrees of freedom, e.g., dispersive elements for spectral modes or diffractive elements for orbital angular momentum, other types of quantum states can be characterized as well (including multiphoton quantum states via n-fold coincidences). Our results thus extend conventional imaging to the quantum domain, providing a pathway for quantum phase retrieval and coherence/entanglement control, and enable new means of quantum information processing with high-dimensional entangled states.

## Methods

The EMCCD (iXon Ultra 897, Andor) is a highly sensitive camera in which an avalanche gain of up to 1000 amplifies the signal in each pixel before readout. The camera has a pixel size of 16 × 16 μm^2^ with a quantum efficiency of ~70% at 800 nm. To minimize the dark-count rate compared to other noise sources in the camera, it is operated at a temperature of −85 °C. The camera is first characterized by measuring the histogram of the gray scale output of each pixel from many (~10^6^) frames taken with the shutter closed. The histogram is primarily Gaussian, due to read noise, with an additional exponential tail towards high gray levels due primarily to clock-induced charge (CIC) noise^34^. We fit the histogram with a Gaussian distribution to find the center (~170) and standard deviation $\sigma$ (4 to 20, depending on the readout rate). We have found that a threshold set to 2*σ* above the mean maximizes the signal-to-noise ratio. A pixel-dependent threshold is used to account for a minor inhomogeneity across the frame. There is a small cross-talk effect between pixels in a single column due to sub-optimal charge transfer efficiency upon readout (see Supplementary Information). For this reason, within each 2D frame of ${{\rm{\Gamma }}}_{i|j}$, we set to zero the 10 pixels above and below $i$ = $j$.

Operating at higher readout rate increases noise from readout and CIC, but we have found that the increased acquisition speed more than compensates, yielding a higher signal-to-noise ratio (SNR) for the same total acquisition time. The camera is therefore operated at the fastest available settings: a horizontal readout rate of 17 MHz and a vertical shift time of 0.3 μs, with a vertical clock voltage of +4 V over factory default. The pump laser power and camera exposure time are set to give an optimum peak count probability $\langle C\rangle$ of ~0.2^34^. We acquire a number of frames sufficient to achieve the desired SNR. Typically, a series of ~10^5^–10^7^ images are acquired at a ~1–5 ms exposure time. Many sets of thresholded frames are saved to disk, where each set contains 10^4^ frames as a logical array ${C}_{i,n}$. Each column of the array represents a single frame, and each row represents a pixel. Equation (2) is used to calculate $\langle {C}_{ij}\rangle$ by matrix multiplication of each set of frames, which are then averaged. To minimize non-ergodic effects, the term $\langle {C}_{i}\rangle \langle {C}_{j}\rangle$ in Eq. (4) is calculated via matrix multiplication of successive frames (see Supplementary Information). Elsewhere, $\langle {C}_{i}\rangle$ is the average of all frames.

In general, the biphoton wave function in an image plane is given by

6 $${\psi }_{img}({{\boldsymbol{\rho }}}_{i},{{\boldsymbol{\rho }}}_{j})={\iint }^{}h({{\boldsymbol{\rho }}}_{i}-{{\boldsymbol{\rho }}^{\prime} }_{i})h({{\boldsymbol{\rho }}}_{j}-{{\boldsymbol{\rho }}^{\prime} }_{j})\cdot t({{\boldsymbol{\rho }}^{\prime} }_{i})t({{\boldsymbol{\rho }}^{\prime} }_{j}){\psi }_{s}({{\boldsymbol{\rho }}^{\prime} }_{i},{{\boldsymbol{\rho }}^{\prime} }_{j})d{{\boldsymbol{\rho }}^{\prime} }_{i}{\rm{d}}{{\boldsymbol{\rho }}^{\prime} }_{j}$$

where ${\psi }_{s}({{\boldsymbol{\rho }}}_{i},{{\boldsymbol{\rho }}}_{j})$ is the wave function incident on the object. With ideally correlated photon pairs, i.e., ${\psi }_{s}({{\boldsymbol{\rho }}}_{i},{{\boldsymbol{\rho }}}_{j})=\delta ({{\boldsymbol{\rho }}}_{i}-{{\boldsymbol{\rho }}}_{j})$, the square amplitude of Eq. (6) simplifies to Eq. (5). The high-resolution biphoton image therefore lies within ${{\rm{\Gamma }}}_{ii}$, where both entangled photons hit the same pixel. However, as EMCCDs are not photon-number-resolving, they cannot distinguish between one or both photons hitting the same pixel. Instead, we approximate ${{\rm{\Gamma }}}_{ii}$ by the case where the two entangled photons arrive in adjacent pixels, i.e., ${{\rm{\Gamma }}}_{i,i+1}$, as we do in Fig. 3f. This assumption is valid when the biphoton correlation width and image features are both larger than the pixel size.

For ideal imaging ($h({\boldsymbol{\rho }})$ ≈ $\delta ({\boldsymbol{\rho }})$), intensity images are directly proportional to ${|t({\boldsymbol{\rho }})|}^{2}$, where $t({\boldsymbol{\rho }})$ is the complex (field) function for transmission. For entangled-photon images,$\,{\rm{\Gamma }}({\boldsymbol{\rho }},{\boldsymbol{\rho }})$ ∝ ${|t({\boldsymbol{\rho }})|}^{4}$ (see Eq. (5)). Therefore, we show in Fig. 3f the the square root of the biphoton images, which is proportional to ${|t({\boldsymbol{\rho }})|}^{2}$, to allow fair comparison to intensity measurements in Fig. 3g,h. This also explains the relative “flatness” of Fig. 3f compared to 3g (which are both computed from the same set of image frames).

## Electronic supplementary material

Supplementary Information
