# Supplementary material for: Massively Parallel Coincidence Counting of High-Dimensional Entangled States
Source: Sci Rep. 2018 May 21;8:7925. doi: 10.1038/s41598-018-26144-7 (PMC5962546; doi:10.1038/s41598-018-26144-7)
Supplement: Supplementary file 1 — Supplementary Information [file 41598_2018_26144_MOESM1_ESM.docx]

Supplemental Information for

**Massively Parallel Coincidence Counting of High-Dimensional Entangled States**

Matthew Reichert*, Hugo Defienne, and Jason W. Fleischer†

Department of Electrical Engineering, Princeton University, Princeton, NJ 08544, USA

E-mail: *mr22@princeton.edu, †jasonf@princeton.edu

**This PDF file includes:**

Supplemental Text

Supplemental Figures

Supplemental References

1. **Biphoton Joint Probability Distribution**

Experimentally, there are two possible ways of measuring entangled photon pairs with detector arrays: (a) photons from pairs are deterministically separated to different detector arrays (or different regions of a single array), and (b) photons are all sent to a single detector array. The principle difference is that both photons from a single pair may hit the same pixel in (b) but not in (a). Equations for the two cases are presented below in tandem, and labeled a and b accordingly, along with equations shared by both cases. Note that in the following we omit the quantum efficiency $\eta$ for brevity. To incorporate it, make the substations $\Gamma_{i}\to\eta\Gamma_{i}$ and $\Gamma_{ij}\to\eta^{2}\Gamma_{ij}$.

The singles count probability at pixel $i$ is given by

|  | $\left\langle C_{i} \right\rangle=\sum_{m} P_{m}\left( \mu_{i\vert m}+p_{el}\mu_{\bar{i}\vert m} \right),$ | (S1) |
| --- | --- | --- |

where $P_{m}$ is the probability distribution for the number of generated pairs and $p_{el}$ is the electronic count probability of the detector (dark counts, CIC, etc.). $\mu_{i|m}$ is the conditional probability, given $m$ photon pairs, that *at least* one photon is detected in pixel $i$. $p_{el}\mu_{\bar{i}|m}$ is the probability of electronic noise counts (counts not due to photons), which requires the absence of detected photons. The factor $\mu_{\bar{i}|m}$ is the conditional probability, given $m$ photon pairs, that no photons are detected in pixel $i$ (indicated by the barred $i$), which is related to the marginal distribution by

|  | $\mu_{\bar{i}\vert m}=\left( 1-\Gamma_{i} \right)^{m},$ $\mu_{\bar{i}\vert m}=\left( 1-\left( 2\Gamma_{i}-\Gamma_{ii} \right) \right)^{m}.$ | (S2a)  (S2b) |
| --- | --- | --- |

In case (a), only one photon from the pair is sent to the detector array of mode $i$, while in case (b) both photons from the pair go to the same array. Multiplying $\Gamma_{i}$ by 2 accounts for this, but $\Gamma_{i}$ also includes the case where the other photon is also in mode $i$, i.e., $\Gamma_{ii}$. Doubling $\Gamma_{i}$ double counts this occurrence, and therefore we must subtract off the extra factor of $\Gamma_{ii}$ in Eq. (S2b).

Because $\mu_{i|m}$ and $\mu_{\bar{i}|m}$ sum to unity, they are related by

|  | $\mu_{i\vert m}=1-\mu_{\bar{i}\vert m}.$ | (S3) |
| --- | --- | --- |

For a Poissonian number distribution of pairs, $P_{m}=\left\langle m \right\rangle^{m}e^{-\left\langle m \right\rangle}/m!$, where $\left\langle m \right\rangle$ is the mean number of photon pairs emitted within exposure time $\tau_{e}$^1,2^, Eq. (S1) simplifies to

|  | $\left\langle C_{i} \right\rangle=1-\left( 1-p_{el} \right)e^{-\left\langle m \right\rangle\Gamma_{i}},$ $\left\langle C_{i} \right\rangle=1-\left( 1-p_{el} \right)e^{-\left\langle m \right\rangle\left( 2\Gamma_{i}-\Gamma_{ii} \right)}.$ | (S4a)  (S4b) |
| --- | --- | --- |

The coincidence count probability between pixels $i$ and $j$ is

| $\left\langle C_{ij} \right\rangle=\sum_{m} P_{m}\left( \mu_{ij\vert m}+p_{el}\left( \mu_{i\bar{j}\vert m}+\mu_{\bar{i}j\vert m} \right)+p_{el}^{2}\mu_{\bar{i}\bar{j}\vert m} \right),$ | (S5) |
| --- | --- |

where the first term represents the probability of coincidence between two photons, the second between one photon and one electronic noise event, and the third between two noise events. As before, the sum of the $\mu$’s is unity: $\mu_{ij|m}+\mu_{i\bar{j}|m}+\mu_{\bar{i}j|m}+\mu_{\bar{i}\bar{j}|m}$ = 1. Coincidences between two electronic noise events depend on photon detections in either pixel $i$ or $j$, which is given by^3^

|  | $\mu_{\bar{i}\bar{j}\vert m}=\left( 1-\Gamma_{i}-\Gamma_{j}+\Gamma_{ij} \right)^{m},$ $\mu_{\bar{i}\bar{j}\vert m}=\left( 1-\left( 2\Gamma_{i}-\Gamma_{ii} \right)-\left( 2\Gamma_{j}-\Gamma_{jj} \right)+2\Gamma_{ij} \right)^{m}.$ | (S6a)  (S6b) |
| --- | --- | --- |

Coincidence counts between photons and electronic noise requires *at least* one photon detection in one pixel and zero in the other. This is given by the probability that no photons are detected in one pixel, i.e., $\mu_{\bar{j}|m}$, minus the probability that no photons are detected in either pixel, $\mu_{\bar{i}\bar{j}|m}$, that is

|  | $\mu_{i\bar{j}\vert m}=\mu_{\bar{j}\vert m}-\mu_{\bar{i}\bar{j}\vert m},$ | (S7) |
| --- | --- | --- |

and vice-versa for $\mu_{\bar{i}j|m}$. The probability that *at least* one photon is detected in each pixel $i$ and $j$ is then

|  | $\mu_{ij\vert m}=1-\mu_{\bar{i}\vert m}-\mu_{\bar{i}\vert m}+\mu_{\bar{i}\bar{j}\vert m}.$ | (S8) |
| --- | --- | --- |

For a Poissonian number distribution of generated pairs, Eq. (S5) becomes

| $\left\langle C_{ij} \right\rangle=1-\left( 1-p_{el} \right)\left( e^{-\left\langle m \right\rangle\Gamma_{i}}+e^{-\left\langle m \right\rangle\Gamma_{j}} \right)+\left( 1-p_{el} \right)^{2}e^{-\left\langle m \right\rangle\left( \Gamma_{i}+\Gamma_{j}-\Gamma_{ij} \right)},$ $\left\langle C_{ij} \right\rangle=1-\left( 1-p_{el} \right)\left( e^{-\left\langle m \right\rangle\left( 2\Gamma_{i}-\Gamma_{ii} \right)}+e^{-\left\langle m \right\rangle\left( 2\Gamma_{j}-\Gamma_{jj} \right)} \right)+\left( 1-p_{el} \right)^{2}e^{-\left\langle m \right\rangle\left( \left( 2\Gamma_{i}-\Gamma_{ii} \right)+\left( 2\Gamma_{j}-\Gamma_{jj} \right)-2\Gamma_{ij} \right)}.$ | (S9a)  (S9b) |
| --- | --- |

Eqs. (S4) and (S9) can thus be used to solve for $\Gamma_{ij}$:

|  | $\Gamma_{ij}=\alpha\ln\left[ 1+\frac{\left\langle C_{ij} \right\rangle-\left\langle C_{i} \right\rangle\left\langle C_{j} \right\rangle}{\left( 1-\left\langle C_{i} \right\rangle\right)\left( 1-\left\langle C_{j} \right\rangle\right)} \right],$ | (S10) |
| --- | --- | --- |

where

|  | $\alpha=\frac{1}{\left\langle m \right\rangle\eta^{2}},$ $\alpha=\frac{1}{2\left\langle m \right\rangle\eta^{2}}.$ | (S11a)  (S11b) |
| --- | --- | --- |

Therefore, to within a constant scaling factor $\alpha$, only the mean coincidence- and singles-count probabilities are necessary to uniquely extract the joint probability distribution.

1. **Residual Background**

In principle, the quantity $\left\langle C_{i} \right\rangle\left\langle C_{j} \right\rangle$ may be calculated as the product of the average of all collected frames. However, we have found that doing so results in a residual background that is not due to genuine coincidence counts. For example, the black data in Fig. S1 shows the momentum anti-correlation measurement in the far-field when the term $\left\langle C_{i} \right\rangle\left\langle C_{j} \right\rangle$ in Eq. (4) is given by

|  | $\left\langle C_{i} \right\rangle\left\langle C_{j} \right\rangle=\left( \frac{1}{N}\sum_{n=1}^{N} C_{i,n} \right)\left( \frac{1}{N}\sum_{n^{'}=1}^{N} C_{j,n^{'}} \right)$ | (S12) |
| --- | --- | --- |

However, since these ensemble averages are calculated via temporal averages, the measurements are susceptible to non-ergodicity. In addition to potential fluctuations in pump laser power, we believe this is due to long-term fluctuation of the gain of the EMCCD camera.

The probability of getting a gray level above threshold, $P\left( x>T | k \right)$ (the detector response function), depends on the input number of photoelectrons $k$, as well as on the noise and gain properties of the camera. The coincidence count distribution may be written

|  | $P\left( x_{i}>T,x_{j}>T\vert k_{i},k_{j} \right),$ | (S13) |
| --- | --- | --- |

which is the probability of getting gray levels above threshold at both pixels $i$ and $j$ given $k_{i}$ and $k_{j}$ photoelectrons at the inputs. (Note that this function depends on the gain of the EMCCD camera^4,5^.) If the pixels are not correlated, then the conditional probability factorizes:

|  | $P\left( x_{i}>T,x_{j}>T\vert k_{i},k_{j} \right)=P\left( x_{i}>T \vert k_{i} \right)P\left( x_{j}>T \vert k_{j} \right).$ | (S14) |
| --- | --- | --- |

Long-time fluctuations of the gain cause variations in the conditional probability distribution over the course of data acquisition, which means that the temporal average does not factorize.

If we instead approximate $\left\langle C_{i} \right\rangle\left\langle C_{j} \right\rangle$ as the product of one frame with the next, i.e.,

|  | $\left\langle C_{i} \right\rangle\left\langle C_{j} \right\rangle\approx\frac{1}{N-1}\sum_{n=1}^{N-1} C_{i,n}C_{j,n+1},$ | (S15) |
| --- | --- | --- |

then the residual background is nearly eliminated, (black data in Fig. S1c). This approximation is justified by the fact that successive frames do not contain photons from the same pair. That is, the exposure plus readout time is sufficiently long—orders of magnitude larger than the biphoton correlation time—that genuine coincidences between pairs of photons only ever occur within a single frame and never between different frames. The fact that the residual background is eliminated indicates that the fluctuations in the gain and/or laser pump power are on much longer time scales compared to the frame rate.


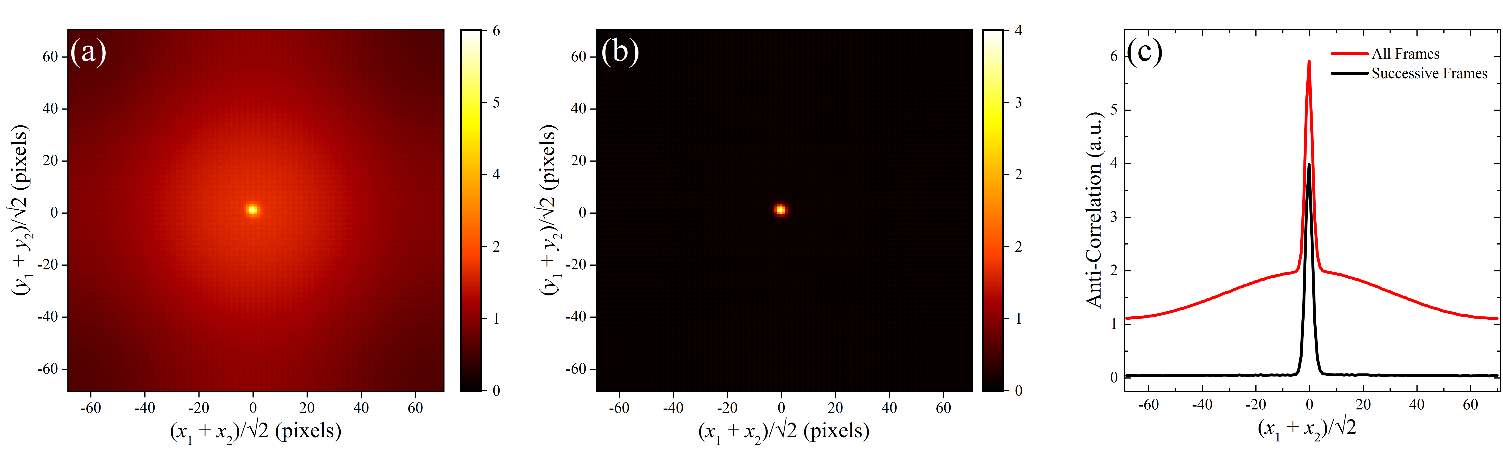


**Fig. S1 | Residual background elimination.** Measurement of momentum anti-correlation calculated via Eq. (4) from main text, where $\left\langle C_{i} \right\rangle\left\langle C_{j} \right\rangle$ is calculated from (a) product of the mean of all frames— Eq. (S12) —and (b) mean of product of successive frames—Eq. (S15). (c) Lineouts at $\left( y_{1}+y_{2} \right)/\sqrt{2}=0$ from (a) (red) and (b) (black), respectively.

1. **Comparison to raster scanning**

The detector array size of an Andor iXon Ultra 888 is 1024 × 1024 pixels, which would correspond to a joint Hilbert space of (1024 × 1024)^2^ ≈ 1 trillion dimensions. While SPCMs have a high effective frame rate (10s of MHz), the acquisition time of an EMCCD camera is practically limited by the readout process. For the camera used here, a definitive speed advantage is found for $d^{2}$ > (24 $\times$ 24)^2^ ≈ 330,000. This particular camera can operate at 26 frames per second when acquiring the entire frame^6^. In far-field measurements of the 4D $\Gamma_{ij}$ with a correlation with $\sigma_{+}$ of 1.2 pixels, we have demonstrated a signal-to-noise ratio (SNR) of 10 with as few as 10^6^ frames. At this SNR, we can measure a 2^40^-dimensional joint Hilbert space in 11 hours.

To compare with raster-scanning single-photon-counters, we first assume ideal conditions (negligible dark count rate and unit quantum efficiency), and determine the number of “frames” needed to achieve the same SNR = 10 for the same $\Gamma_{ij}$ as above. We first solve

|  | $\mathrm{SNR}=\frac{\sqrt{N}}{2\pi\sigma_{+}^{2}},$ | (S16) |
| --- | --- | --- |

and find $N$ = 8187. The acquisition time depends on the dead time of the photon counting modules, whose inverse gives the effective frame rate. For a typical single-photon-counting module (SPCM-AQRH Series, Excelitas Technologies^7^) the output count rate before saturation, i.e., the effective frame rate, is $R_{f}$ = 37 MHz. Given sufficient biphoton flux, it therefore takes $N/R_{f}$ = 220 μs to acquire enough frames to achieve the desired SNR. This, however, must be repeated over the entire trillion-dimensional joint Hilbert space. In practice, we may take advantage of the fact that the biphoton joint probability distribution is symmetric upon exchange, and reduce the number of measurements down to half a trillion. Therefore, measurement with raster-scanning point detectors would take at least (220 μs)(1024^4^/2) ≈ 3.85 years. For more realistic conditions—taking the quantum efficiency of 0.65 into account^7^—this number increases to 9.14 years. This also assumes the raster scanning is limited only by the acquisition time at each position, and that the time to translate between points is negligible.

1. **Effects of Charge Transfer Inefficiency**

During the readout process of the EMCCD camera, charge is transferred vertically through columns of the array to the readout register. This process does not occur with 100 % efficiency, i.e., the probability to transfer all the electrons from one pixel to the next less than unity. This concept is quantified by the Charge Transfer Efficiency (CTE), which may be very close to one (typically in excess of 0.9999996^8^). Defect states in individual pixels are responsible for trapping charge, resulting in signal loss at the pixel of interest and vertical smearing. This effect has been studied extensively, particularly in the astronomy community, where ionization and bulk damage is caused by high-energy photons and particles in spacecraft^8-11^.


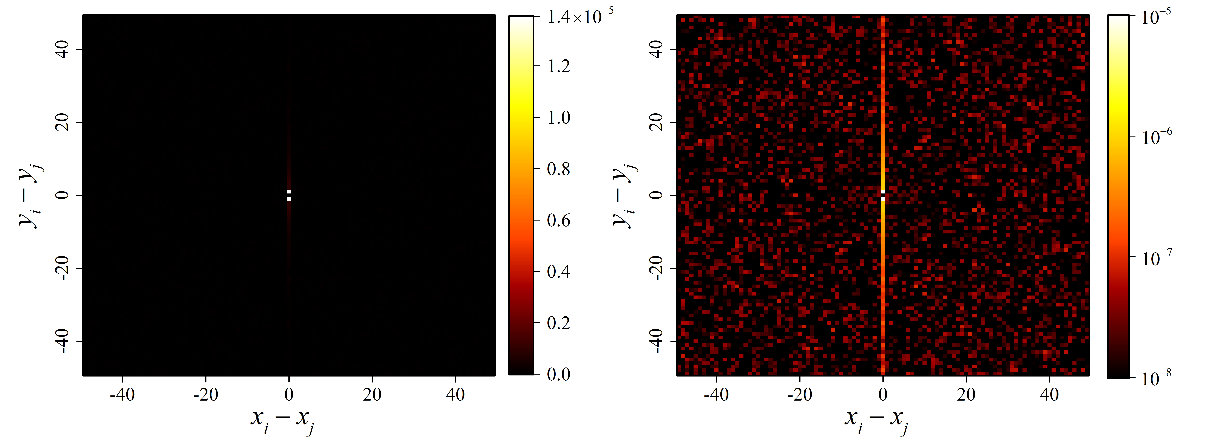


**Fig. S2 | Projection of** $\boldsymbol{\Gamma}_{\boldsymbol{ij}}$ **onto difference coordinates** $\boldsymbol{[}\boldsymbol{x}_{\boldsymbol{i}}\boldsymbol{-}\boldsymbol{x}_{\boldsymbol{j}}\boldsymbol{,}\boldsymbol{y}_{\boldsymbol{i}}\boldsymbol{-}\boldsymbol{y}_{\boldsymbol{j}}]$**.** Data from 3×10^6^ thresholded frames of (99 × 99) pixels measured with the camera shutter closed on a (a) linear and (b) logarithmic color scale. Pixel at [0, 0] is set to zero. Vertical line at $x_{i}-x_{j}$ = 0 is due to imperfect vertical charge transfer during readout, resulting in artificial correlation between pixels in the same column.

This cross talk effect results in a correlation between one pixel and those in the same column, particularly in those immediately above and below. An example is shown Fig. S2, where 3×10^6^ thresholded frames were measured with the camera shutter closed, such that all registered “clicks” originated from electronic noise. From these frames, $\Gamma_{ij}$ was calculated via Eq. (4) and projected onto difference coordinates. Nominally, since no biphotons reach the camera, the result should be zero. However, we clearly see a correlation, particularly between one pixel and the pixels directly above and beneath.

1. **References**

1 Larchuk, T. S., Teich, M. C. & Saleh, B. E. A. Statistics of Entangled-Photon Coincidences in Parametric Downconversion. *Annals of the New York Academy of Sciences* **755**, 680-686, (1995).

2 Avenhaus, M. *et al.* Photon Number Statistics of Multimode Parametric Down-Conversion. *Physical Review Letters* **101**, 053601, (2008).

3 Tasca, D. S., Edgar, M. P., Izdebski, F., Buller, G. S. & Padgett, M. J. Optimizing the use of detector arrays for measuring intensity correlations of photon pairs. *Physical Review A* **88**, 013816, (2013).

4 Lantz, E., Blanchet, J.-L., Furfaro, L. & Devaux, F. Multi-imaging and Bayesian estimation for photon counting with EMCCDs. *Monthly Notices of the Royal Astronomical Society* **386**, 2262-2270, (2008).

5 Basden, A. G., Haniff, C. A. & Mackay, C. D. Photon counting strategies with low-light-level CCDs. *Monthly Notices of the Royal Astronomical Society* **345**, 985-991, (2003).

6 *Andor iXon Ultra EMCCD Specifications*, <http://www.andor.com/pdfs/specifications/Andor_iXon_ULTRA_EMCCD_Specifications.pdf>

7 *SPCM-AQRH Single Photon Counting Module*, <https://www.pacer-usa.com/Assets/User/2072-SPCM-AQRH.pdf>

8 Janesick, J. R., Soli, G. B., Elliott, T. S. & Collins, S. A. in *Proc. SPIE 1441. Charge-Coupled Devices and Solid State Optical Sensors II.* 87-108 (SPIE).

9 Goudfrooij, P., Bohlin, R. C., Maíz‐Apellániz, J. & Kimble, R. A. Empirical Corrections for Charge Transfer Inefficiency and Associated Centroid Shifts for STIS CCD Observations. *Publications of the Astronomical Society of the Pacific* **118**, 1455, (2006).

10 Daigle, O. *et al.* Extreme Faint Flux Imaging with an EMCCD. *Publications of the Astronomical Society of the Pacific* **121**, 866, (2009).

11 Chiaberge, M. A new accurate CTE photometric correction formula for ACS/WFC. *ACS/ISR* **2015**, (2012).
